# Key challenges in neurocognitive assessment of individuals with antisocial personality disorder and psychopathy

**DOI:** 10.3389/fnbeh.2022.1007121

**Published:** 2022-09-02

**Authors:** Julia Griem, Nathan J. Kolla, John Tully

**Affiliations:** ^1^Department of Forensic and Neurodevelopmental Sciences, Institute of Psychiatry, Psychology, and Neuroscience, King's College London, London, United Kingdom; ^2^Department for Psychiatry, University of Toronto, Toronto, ON, Canada; ^3^Research Imaging Centre, Centre for Addiction and Mental Health (CAMH), University of Toronto, Toronto, ON, Canada; ^4^Research and Academics, Division, Waypoint Centre for Mental Health Care, Penetanguishene, ON, Canada; ^5^Academic Unit of Mental Health and Clinical Neurosciences, School of Medicine, University of Nottingham, Nottingham, United Kingdom

**Keywords:** neurocognitive assessment, neuropsychological tests, antisocial personality disorder, psychopathy, challenges

## Introduction

Individuals with conduct disorder (CD) in youth and antisocial personality disorder (ASPD) in adulthood are responsible for a large proportion of violent crime (Falk et al., 2014; Martinez et al., 2017). Within this group, about one-third meet criteria for callous-unemotional (CU) traits in youth and psychopathy (ASPD+P) in adulthood, offending earlier, more widely, and more severely than those without psychopathy (ASPD-P) (Kosson et al., 2006). Despite the high prevalence of ASPD+/-P in forensic and penal settings (Fazel and Danesh, 2002; Coid et al., 2009b) and the large social and financial burden associated with ASPD+/-P (Heeks et al., 2018), evidence for successful treatments is lacking (Gibbon et al., 2020; Khalifa et al., 2020), and understanding of causative mechanisms remains limited.

The identification of causative mechanisms relating to neurocognitive factors that contribute to the development of ASPD and its heterogeneity may be particularly important. Studies have begun to offer insight. This includes evidence for difficulties in emotion recognition and empathic responding (Blair, 2007; Marsh and Blair, 2008; Dawel et al., 2012; Decety et al., 2013; Schönenberg and Jusyte, 2014), reinforcement-based learning (Budhani et al., 2006; Dolan, 2012; De Brito et al., 2013; Gregory et al., 2015; Hughes et al., 2016; Glimmerveen et al., 2022a), and attention (Hamilton and Newman, 2018; Baliousis et al., 2019; Baskin-Sommers and Brazil, 2022). Improved knowledge of the underpinnings of these dysfunctions could help identify important treatment targets and lead to the development of stratified, focused interventions, the potential for which has been previously demonstrated (Baskin-Sommers et al., 2015).

Despite this promise, there are several important challenges which constrain the potential of neurocognitive testing in ASPD and psychopathy. Key amongst these are (1) inconsistent phenotypic characterization of heterogeneous samples; (2) unreliable/inconsistent task design and selection; (3) poor task engagement; and (4) the lack of longitudinal studies. Below, we discuss these in turn and make suggestions for optimization of future research.

## Key challenges in neurocognitive assessment in ASPD+/-P

### Inconsistent phenotypic characterization

While some dimensional understanding of ASPD is important, a pragmatic approach to addressing heterogeneity of ASPD is to stratify them into more biologically homogenous subtypes (Brazil et al., 2018). Together with the divergent offending profiles of ASPD+/-P, accumulating evidence suggests that individuals with ASPD+P compared to those with ASPD-P have shared but also distinct neurobiological/neurocognitive features (Kosson et al., 2006; Gregory et al., 2012, 2015; De Brito et al., 2013; Pera-Guardiola et al., 2016; Marsden et al., 2019).

Such stratification, however, requires a consistent definition of psychopathy. The Psychopathy Checklist-Revised (PCL-R) is the most widely used assessment tool in clinical forensic and penitential populations (Hare, 1991), but considerable debate persists about the most appropriate construct of psychopathy (Cooke and Michie, 2001; Hare and Neumann, 2008). This has led to the use of other tools including self-report questionnaires, which may have lower reliability, since individuals with ASPD+/-P might not tell the truth or have enough insight (Brinkley et al., 2001; Sellbom et al., 2007; Gonsalves et al., 2013). The assessment of community-dwelling individuals with subclinical psychopathic traits also limits the ability to form a consistent understanding of psychopathy (e.g., Esser and Eisenbarth, 2021; Friedman et al., 2021). Considering the prevalence of subclinical psychopathic traits in the community (Coid et al., 2009a), this is not problematic per se. However, it is questionable whether such findings are applicable to clinical samples, where more severe neurocognitive deficits might have different underpinnings. Finally, even studies of clinical psychopathy which use the PCL-R choose different cut-off points. Evidence suggests that a cut-off point of 25 should be used for European samples (as opposed to 30 for US samples) (Cooke and Michie, 1999), likely due to cultural factors, however this is not always adhered. Furthermore, some studies might refer to their sample as “high” on psychopathy despite scores only indicating subclinical levels of psychopathy (e.g., Domes et al., 2013; Weidacker et al., 2017).

Together, these inconsistencies contribute to incongruent results in neurocognitive assessments, which complicates the development of clear neuropsychological models of ASPD+/-P. Ongoing research into the most appropriate construct of psychopathy remains of importance. However, perhaps the time has come to develop a large-scale collaborative protocol that agrees on the most appropriate tools to measure psychopathy within clinical forensic research. This would not preclude using multiple metrics of psychopathy. Indeed, sufficiently well-powered, pre-registered neurocognitive studies would allow for meaningful investigation into which constructs correlate best with potential biomarkers.

### Unreliable/inconsistent task design and selection

For any given neurocognitive function, there are myriad tasks claiming to provide reliable metrics. However, these often slightly vary from one another. This leads to problems when comparing or collating findings and may partially explain inconsistent or contradictory results found in ASPD+/-P samples that are otherwise similar (Griffiths and Jalava, 2017). A key example is empathy deficits. Despite being a clinical feature of ASPD (First et al., 2015) and a critical component of the psychopathy construct (Hare, 1991), a recent systematic review found that ASPD+/-P was not associated with deficits in neurocognitive paradigms of empathy (Marsden et al., 2019). While underpowered studies in a population where recruitment is difficult may be a factor, it is also likely that inconsistency in task design and selection plays a role. Empathy is a broad concept with many facets. Neurocognitive research of empathy suggests several underpinning neurocognitive mechanisms (Bird and Viding, 2014), however there are competing theories about how it is best conceptualized (see Decety and Ickes, 2011; Zaki and Ochsner, 2012; Blair, 2018). The most common distinction is made between cognitive empathy (including mentalizing and theory of mind) and affective empathy (including experience sharing and emotional contagion). However, even when studies attempt greater specificity, for example by reference to mentalization, different researchers may in fact be referring to subtly different neurocognitive functions (Choi-Kain and Gunderson, 2008). This complicates the development and interpretation of tasks measuring empathy.

Evidence suggests some shared, but some distinct elements of empathic profiles in ASPD+/-P. Adults with ASPD+/-P show relatively normal performance on some aspects of cognitive empathy (Blair et al., 1996; Dolan and Fullam, 2004; Shamay-Tsoory et al., 2010), though those with ASPD+P fail to automatically take others' perspective (Drayton et al., 2018). In contrast, individuals with ASPD+/-P appear to diverge in key aspects of affective empathy. Studies indicate that brain regions involved in implicit responsivity to others' pain and emotional faces may be *hypo*responsive in ASPD+P (Decety et al., 2013, 2014; Contreras-Rodríguez et al., 2014). Contrarily, individuals with ASPD-P are thought to be *hyper*responsive to emotional stimuli, particularly fear and threat (Schönenberg and Jusyte, 2014; Hodgins et al., 2018). Hence, disentangling the relative contributions of deficits in cognitive and affective components of empathy by using consistent and reliable task designs may be crucial in developing treatments for specific deficits. The recent development of EmpaToM, a validated tool which delineates cognitive and affective empathy, is a step in the right direction (Kanske et al., 2015). This has already been used to demonstrate deficits in affective empathy, but not theory of mind, in male violent offenders (Winter et al., 2017).

### Poor task engagement

Poor attention, lack of interest or motivation to participate in activities which are not self-beneficial, irresponsibility, and propensity for lying are all inherent pathological features of ASPD, and particularly, psychopathy. These features complicate assessment procedures and could lead to false positive and false negative findings. For instance, a lack of effort due to poor motivation could lead to significantly poorer performance on common outcome measures such as accuracy and reaction times than would be expected in real-life scenarios. In contrast, false negative findings could emerge due to unreliable performance. Individuals with psychopathy, where pathological lying is a feature, may be prone to report inaccurate responses, not reflecting their true emotional reaction, in tasks measuring explicit responses to emotions.

An example of the interpretation of task findings being complicated by potential poor engagement can be found in a recent fMRI experiment (Tully, 2021), which was designed to capture the neural circuitry of cooperation versus retaliation using the “Dealmaking” game (White et al., 2016). This study included male violent offenders with ASPD-P who were clinically characterized by high reactive aggression and low tolerance to frustration and threat. Based on previous findings in similar populations (White et al., 2013, 2014; **?**), and assuming that their clinical profiles would be reflected in their task-based decision-making, it was predicted that they would decide to “reject and punish” unfair financial offers. Instead, they typically accepted unfair offers and punished less frequently than healthy non-offending controls (though findings were not statistically significant) (Tully, 2021). While it is possible this reflects the absence of neuropsychological differences between ASPD-P and controls, other explanations seem more feasible. It may be that the lack of actual monetary incentives meant subjects were not sufficiently motivated, or that subjects became bored or uninterested, as was reflected in some feedback comments collated separately. The experiment also found no activation of threat circuitry on fMRI, which would support the view that the task did not elicit a cooperation vs. retaliation decision in the ASPD-P group.

Future research may benefit from strategies to mitigate such risks. To disentangle true task effects from the potential impact of inherent clinical symptoms, it may be valuable to include experimental manipulations measuring attention. Furthermore, approaches to increase the motivation and engagement of individuals with ASPD and psychopathy should be considered. For example, a recent study highlighted the importance of adequate and tangible incentives or personalized rewards (Glimmerveen et al., 2022b). One time- and cost-effective solution is the incorporation of control items or performance validity tests (Greher and Wodushek, 2017; Sweet et al., 2021). This can be helpful toward identifying data that should be excluded from analysis due to poor engagement. It is also important that all participants receive the same precise instructions, to avoid different understandings of the task. For example, effort might fluctuate if participants are focusing on fast vs. accurate responses (Ging-Jehli et al., 2021). Lastly, it is important to consider task difficulty. Tasks that are too simple may promote boredom, whereas tasks that are too difficult may hinder effort (Cornacchio et al., 2017).

### Lack of longitudinal studies

Since CD+/-CU traits is the psychopathological developmental precursor of ASPD+/-P, several pivotal large cohort studies have followed the trajectories of youth with CD for up to several decades (Frick and Viding, 2009; Frick et al., 2013; Assink et al., 2015; Jolliffe et al., 2017; Moffitt, 2018; Carlisi et al., 2020; Farrington, 2020; Lasko and Chester, 2021). These have provided crucial insights by identifying early cognitive risk factors for differential pathways toward life-course persistent antisocial behavior and offer improved understanding of protective and promotive factors. However, they have been limited by the relative lack of thorough neurocognitive testing. This means that the understanding of how specific neurocognitive mechanisms change over time is still limited. In contrast research of other neurodevelopmental disorders such as autism has shown that the neurobiological underpinnings may continue to develop differently throughout adulthood, highlighting the importance of longitudinal assessments across the lifespan (Magiati et al., 2014). This may be especially important in ASPD+P, which has many features consistent with neurodevelopmental disorders, including considerable heritability and a male preponderance (Viding et al., 2005; Baron-Cohen et al., 2011; Yildirim and Derksen, 2012). Longitudinal studies could also identify important distinctions in the trajectories of males and females with psychopathy (Tully et al., 2022). In keeping with this view, other authors have identified neuropsychological markers, but also their patterns of change over time, as essential in order to develop personalized medicine approaches (Blair et al., 2022). Encouragingly, large-scale collaborations in the study of antisocial youths have begun to adopt this approach (Casey et al., 2018; Freitag et al., 2018). These important developments align with a wider move toward replicability and transparency in psychological research (Munafò et al., 2017).

## Conclusion

We have highlighted four key issues that continue to limit neurocognitive testing in ASPD+/-P, relating to both psychometric assessment and study methodology. Alongside specific improvements in task design and execution and longitudinal assessments, a recurring theme within potential solutions is a consistent, collaborative approach. Large-scale collaborative studies guided by scientific discourse among experts is required to move toward a personalized medicine approach that uses neurocognitive markers as treatment targets for ASPD+/-P. This is essential if we are to help alleviate the personal, social and financial burden associated with these complex disorders.

## Author contributions

JG and JT were responsible for the conceptualization of the topic and the writing and development of the manuscript. NK provided additional input and editorial support on the manuscript. All authors contributed to the article and approved the submitted version.

## Funding

JG is in receipt of a PhD studentship funded by the National Institute for Health and Care Research (NIHR) Biomedical Research Centre at South London and Maudsley NHS Foundation Trust and King's College London (grant code IS-BRC-1215-20018).

## Conflict of interest

The authors declare that the research was conducted in the absence of any commercial or financial relationships that could be construed as a potential conflict of interest.

## Publisher's note

All claims expressed in this article are solely those of the authors and do not necessarily represent those of their affiliated organizations, or those of the publisher, the editors and the reviewers. Any product that may be evaluated in this article, or claim that may be made by its manufacturer, is not guaranteed or endorsed by the publisher.

## Author disclaimer

The views expressed are those of the author(s) and not necessarily those of the NHS, the NIHR or the Department of Health and Social Care.
